# Light Weight, Easy Formable and Non-Toxic Polymer-Based Composites for Hard X-ray Shielding: A Theoretical and Experimental Study

**DOI:** 10.3390/ijms21030833

**Published:** 2020-01-28

**Authors:** Mattia Lopresti, Gabriele Alberto, Simone Cantamessa, Giorgio Cantino, Eleonora Conterosito, Luca Palin, Marco Milanesio

**Affiliations:** 1 Dipartimento di Scienze e Innovazione Tecnologica, Università degli Studi del Piemonte Orientale, viale T. Michel, 11-15121 Alessandria, Italy; mattia.lopresti@uniupo.it (M.L.); simone.cantamessa@uniupo.it (S.C.); giorgio.cantino@uniupo.it (G.C.); eleonora.conterosito@uniupo.it (E.C.); luca.palin@uniupo.it (L.P.); 2 Bytest s.r.l.-TÜV SÜD Group, Research Center, via Pisa 12, 10088 Volpiano, Italy; gabriele.alberto@tuv.it

**Keywords:** epoxy resins, barium sulfate, bismuth oxide, polymer composite, hard X-ray shielding, life cycle assessment, experimental design, X-ray radiography, Geant4 simulations, physical-chemical characterization

## Abstract

Composite lightweight materials for X-ray shielding applications were studied and developed with the goal of replacing traditional screens made of lead and steel, with innovative materials with similar shielding properties, but lighter, more easily formed and workable, with lower impact on the environment and reduced toxicity for human health. New epoxy based composites additivated with barium sulfate and bismuth oxide were designed through simulations performed with softwares based on Geant4. Then, they were prepared and characterized using different techniques starting from digital radiography in order to test the radiopacity of the composites, in comparison with traditional materials. The lower environmental impact and toxicity of these innovative screens were quantified by Life Cycle Assessment (LCA) calculation based on the ecoinvent database, within the openLCA framework. Optimized mixtures are (i) 20% epoxy/60% bismuth oxide/20% barite, which guarantees the best performance in X-ray shielding, largely overcoming steel, but higher in costs and a weight reduction of *circa* 60%; (ii) 20% epoxy/40% bismuth oxide/40% barite which has slightly lower performances in shielding, but it is lighter and cheaper than the first one and (iii) the 20% epoxy/20% bismuth oxide/60% barite which is the cheapest material, still maintaining the X-ray shielding of steel. Depending on cost/efficiency request of the specific application (industrial radiography, aerospace, medical analysis), the final user can choose among the proposed solutions.

## 1. Introduction

X-ray shielding is particularly interesting for many applications, from the industrial field of non destructive control techniques (radiography and tomography), airport scanners and control [1] to the medical field (radiology and dentistry) [2,3]. Nowadays the most common shields for X-rays are still made of lead and steel. While lead has excellent shielding properties, its mechanical features are very poor, being very soft and malleable. Steel, instead, has sufficient shielding properties and excellent mechanical performances but high costs and limited workability for the realization of shielding masks by machining. These materials have complementary advantages in the quality of the shielding, soft X-rays cutting and good mechanical properties, but the drawbacks are objects weight, human health hazards and environment pollution. The principal solutions given in the specialized literature are based on three different types of materials: inorganic, organic and composites. The most common alternative to the traditional shielding is the use of inorganic materials: a compressed slab of minerals like barium sulfate (BaSO_4_), calcium sulfate (CaSO_4_) or calcium carbonate (CaCO_3_). This kind of solution is easy to obtain but the objects have very poor mechanical properties, with low tensile strength and tendency to crumble. Furthermore, the weight gain is not so convenient. The second solution is represented by organic materials in which the number of electrons by volume unit is increased by adding iodine atoms to polymeric chains: mostly, isoprenic rubber is synthesized with monomers that have iodine atoms covalently bound. Examples of these monomers are iopanoic acid 3-(3-amino-2,4,6-triiodophenyl)-2-ethyl propanoic acid and iothalamic acid (5-acetamido-2,4,6-triiodo-N-methyl iso-phthalamic acid) and 2,4,6-triiodobenzoic acid [4,5,6]. Resulting materials have sufficient radiopacity and good mechanical performances. Also, as the iodine is covalently bound, the chances of leaks are low. However, these kinds of shields are unfit for industrial radiography, where the photon energies are larger than 150 keV. In this region, because of the dilution of iodine into the polymer, the shielding power is very low. The last class is that of composite materials, attempting at joining the qualities of minerals (high shielding and no or low toxicity) with those of metals (good mechanical properties), adding an improved workability and lower cost. Composites are divided in two sub-classes:

- metal powders embedded in the polymeric matrices. Some of these solutions are already in commerce, e.g., tungsten-loaded polylactic acid for 3D-Printing [7];
- minerals included in polymeric matrices, typically high density oxides such as zinc oxide (ZnO), tin oxide (SnO_2_) or bismuth oxide (Bi_2_O_3_) [2,8,9,10] and compounds of materials of the sixth period of the periodic table such as barium sulphate (BaSO_4_) [11].

A critical factor for composite materials is dispersion and adhesion of the solid materials to the polymer, in order to avoid both fractures and leakage of heavy transition/post-transition metals. Another critical issue resides in the homogeneous distribution of the additive in the whole volume of the sample, as evidenced by Prolongo et al. [12]. The sedimentation of the powder on the bottom side of the sample, may bring to three effects:

- reduction of the effective thickness of the shielding material, which causes the reduction of the overall shielding performance;
- different mechanical properties in the same sample based only on which side is considered, a chance that could bring to fracture or lower the overall resistance;
- anisotropic shielding yields.

In this paper, we focus on developing low-cost lightweight shielding composite materials that can be easily processed, formed in complex shapes and produced. Materials from scientific literature were simulated with Geant4 to test their screening performances. Commercial epoxidic resins were chosen because of their low cost, easy formability and workability. Barium sulphate and bismuth oxide were chosen because of their good balance of low cost, radiopacity, low environmental impact and absent or low toxicity. Different composite formulations were then predicted with a Simplex-lattice experimental design and developed in laboratory. Materials were fully characterized using XRPD, optic and electronic microscopy, computed radiography and tensile strength test. The overall environmental impacts of the samples were examined through an LCA study “from the cradle to the gate” and compared to the same calculations for the traditional screens of lead and steel. It was thus possible to develop composite formulations able to assure the best performances in term of shielding, weight and low cost depending on final user requests.

## 2. Results

### 2.1. Geant4 Simulations

**Figure 1:**
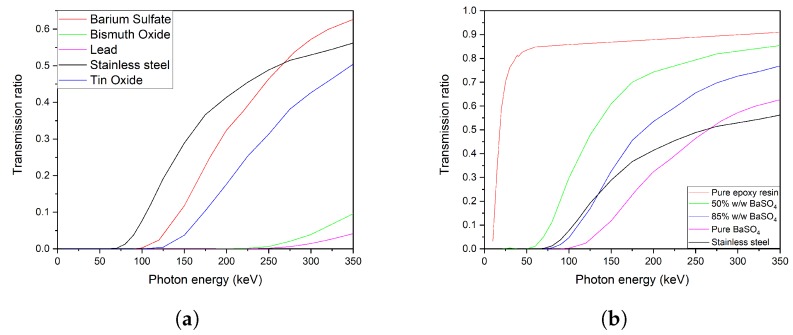
First generation of simulations. These data are referred to 1 cm cubes irradiated with a monochromatic beam of X-rays at successive steps of photon energies, from 0 to 350 keV. In (**a**) common shielding materials from scientific literature are compared. In (**b**) samples of mixture between epoxy resin and BaSO_4_ are simulated and compared to stainless steel. (**a**) Pure materials; (**b**) BaSO_4_ samples.

**Figure 2:**
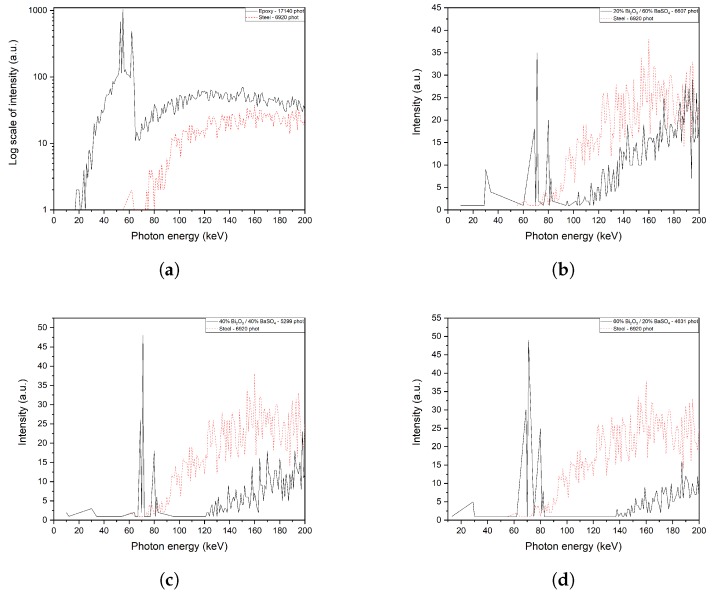
Simulations of the transmitted spectrum of Bremsstrahlung of a tungsten target of 300 μm through a sample of a 1 cm-sided cube of different mixtures compared to the same solid of stainless steel. (**a**) shows the transmitted spectrum through a sample of pure epoxy resin with no additive; the log scale was set due to the high rate of transmitted photons that crossed the polymeric materials. (**a**) Pure epoxy resin; (**b**) 60% BaSO_4_, 20% Bi_2_O_3_, 20% Epoxy; (**c**) 40% BaSO_4_, 40% Bi_2_O_3_, 20% Epoxy; (**d**) 20% BaSO_4_, 60% Bi_2_O_3_, 20% Epoxy.

**Figure A1:**
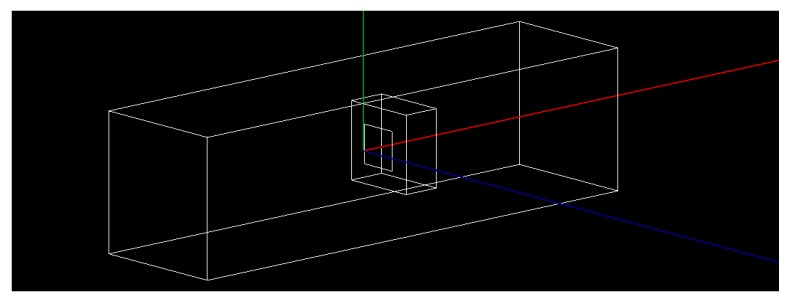
Simulation of shielding properties of the materials. Electrons are generated from one side of the envelope and travel through the space filled with vacuum via ”G4_Galactic” material. Electrons hit the tungsten target, which generates characteristic X-ray emission. This spectrum (the Bremsstrahlung spectrum of W) is focused on the sample, in order to evaluate its radiopacity by measuring the number of the transmitted X-rays.

The first batch of simulations was made with a software based on Geant4 which simulates the interaction between electromagnetic radiation and matter; the interaction of pure materials selected from the scientific literature with monochromatic beams of X-rays was then studied adopting successive steps of energy in the range 0–350 keV, as described in Appendix B. This approach can be defined a *“soft simulation”* because of its approximation, since in industrial radiography the incident beam is a spectrum, more specifically the Bremsstrahlung spectrum plus the characteristic emissions of the anti cathode of the X-ray tube. The aim is estimating which materials were the best fitted for shields that have to be not only radiopaque, but also economically affordable and mechanically resistant. As showed in Figure 1a, the three principal additives from scientific literature, BaSO_4_, Bi_2_O_3_ and ZnO, over-perform the stainless steel in the range 75–240 keV, which is also the main energy of photons of standard X-ray tubes for radiography. Also, simulated samples of composites epoxy resin—BaSO_4_ were compared to steel in order to evaluate the effect that would be obtained by adding this inorganic additive into the epoxy resin (Figure 1b). After this analysis, ZnO was discarded because its shielding properties are similar to those of barium sulfate, but its cost is about 100 times higher. Samples predicted from the Simplex-lattice experimental design for a ternary mixture were simulated in a second time with a second application built with Geant4, which allowed a more accurate approach to the problem. This new simulation setup featured a thin tungsten solid between the beam source and the sample. Incident particles were also changed from X-rays to electrons, so that from the first impact with the anti cathode, a characteristic Bremsstrahlung spectrum of X-rays of W would be generated and focused on the sample, as described in Figure A1 in Appendix B. Figure 2a–d show the simulated behavior of each sample compared to steel. In the range of 30 keV–80 keV some spikes are evident, and are the characteristic emissions of the tungsten target (K${}_{\beta_{1}}=67.24$ keV, K${}_{\beta_{2}}=69.10$ keV) or effects of absorption-emission from the heavy atoms in the screens ( Barium $K_{\alpha_{1}}=31.81$ keV, Barium K${}_{\alpha_{2}}=32.19$ keV, Bismuth K${}_{\alpha_{1}}=74.82$ keV, Bismuth K${}_{\alpha_{2}}=77.11$ keV). Results of this simulation set can be seen in Figure 2. The large noise in the simulation results is due to the high rate in absorption from the material; on the other hand, a fit of the resulting data would lead to a loss of information about the K${}_{\alpha }$ and K${}_{\beta }$ riemissions from the heavy metals in the simulated samples. Studied samples all perform better than steel, in particular sample of Figure 2d.

### 2.2. Setup and Optimization of Sample Preparation Methods

The inorganic additives were analyzed with X-ray Powder Diffraction to evaluate the purity of the minerals before being added to the polymeric matrix. The XRPD patterns of barium sulfate by Itaprochim and Universal Services S.r.l. and of bismuth oxide by Thermo Fischer (Kandel, Germany) were collected. After identifying the impurities by peak matching using QualX [13], Rietveld refinement was performed on the XRPD patterns using structures from the Crystallographic Information Files retrieved from the Crystallographic Open Database and TOPAS Academic V5 [14]. Identified in the first barium sulfate batch (Itaprochim) some impurities: calcite (CaCO_3_), and dolomite (CaMg(CO_3_)_2_) 0.8 and 13.6%w/w respectively. Blanc fixe barium sulfate (Universal Services S.r.l.) and Bi_2_O_3_ showed a purity of 99.5% and no relevant impurities were found by XRPD analysis.

Nine samples (Table 1) were produced with epoxy resin and an increasing mass fraction of BaSO_4_ (Itaprochim), from 30%w/w to 85%w/w according to the procedure described in Section 5 and shown in Appendix C; 85%w/w was observed to be the upper limit of wettability of the powders and, consequently, the limit of mixability between powders and liquid precursors of the epoxy resin. XRPD was also used to characterize this same batch of nine barite samples. After their curing, they were analyzed on both sides: the one exposed to the air and the one in contact with the mold. The samples showed different behaviors depending on the sides and the quantity of inorganic additive.

**Figure 3:**
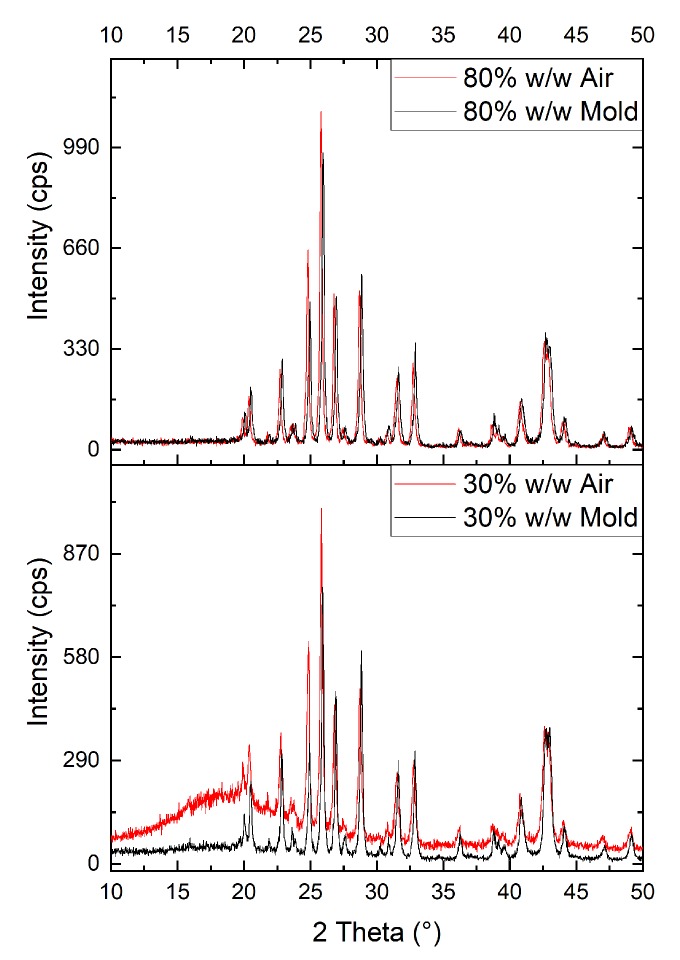
XRPD patterns collected on both sides of sample 2 (30% *w*/*w* in BaSO_4_) and sample 7 (80% in BaSO_4_).

**Figure 4:**
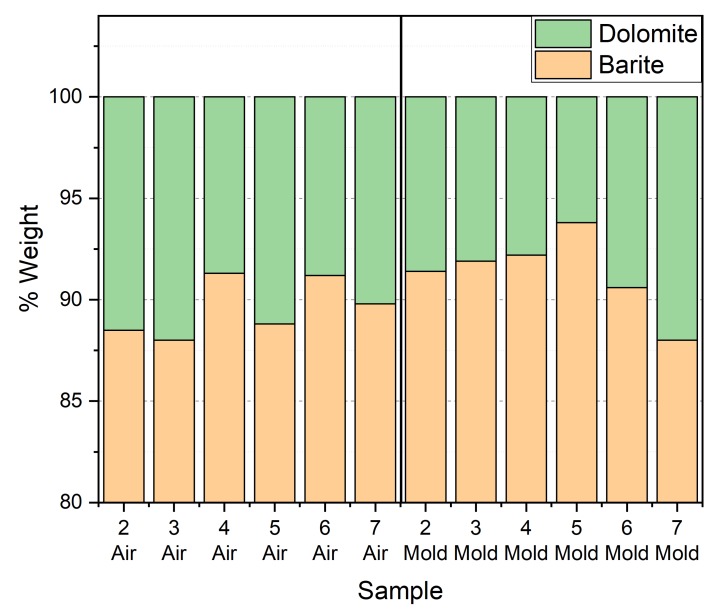
Quantitative analysis of crystalline phases of barite (BaSO_4_) and dolomite (CaMg(CO_3_)_2_) was done on air and mold sides of samples of epoxy/barite.

In Figure 3, bottom graph, in sample 2 the broad band of the amorphous epoxy polymer is evident, suggesting that most of the inorganic moved down in the sample during the curing. On the bottom side of the sample (Mold in Figure 3), the barium sulfate pattern is dominant, confirming the sedimentation of the additive. Rietveld refinement of all datasets indicated a different behaviour of barite and of dolomite impurities. Because of their different density and size, up to sample 5 the sedimentation of barite is larger with an increasing amount in the mold side with respect to dolomite. Conversely in samples 6, 7 and 8 this behavior is not observed and the trend is inverted with a smaller barite percentage in the mold side. This trend can be explained because samples 6, 7 and 8 were already rather viscous before curing and therefore the sedimentation effect was limited (Figure 4). In samples 7 and 8 the amorphous band is not visible and the intensity of air side and mold side patterns are very similar. The refined peak shift confirms the sedimentation hypothesis. A peak shift is observed but it is a combination of the sedimentation and of sample imperfections (bubbles and rugosity), causing a misalignment of the sample. XRPD evidenced a progressive sedimentation process, occurring during the time of gelification of the resin. The phenomenon is more evident in samples with an inorganic fraction smaller than 60%w/w, as the viscosity is low and the powders encounters little resistance to the deposition process.

### 2.3. Experimental Design

**Figure 5:**
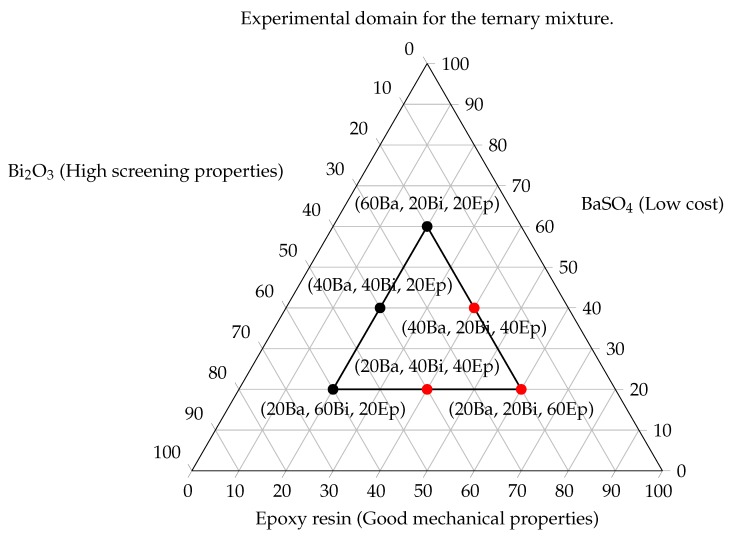
Simplex-lattice design algorithm applied to a mixture for a 3-dimensional experimental domain. To each of the three component of the mixture domain a property was associated: low cost for barite, highest radiopacity for bismuth oxide and high mechanical performances for the epoxy resin. Black dotted samples were developed in laboratory, while red dotted samples were not due to an excessive radiotransparency to hard X-rays.

Sample compositions were selected applying the Simplex-Lattice design algorithm described in Appendix A. The extension of the simplex, therefore the minimum amount of each component of the mixture, was decided considering the miscibility limit for the binary mixture BaSO_4_-epoxy, which was observed as 85%w/w, as discussed in Section 2.2. To avoid using the exact limits of the experimental domain the upper limit on the quantity of additive in the epoxy precursor was set to 80%. Simplex-Lattice design described in Appendix A explores all the experimental domain, which in this case would not be useful due to the fact that the lone resin is completely transparent to X-rays and the other two vertexes of the simplex would lead to two slabs of pure compacted powder with no mechanical properties. Being the minimal content of epoxy resin equal to the 20%w/w of the total weight, the consequent simplex obtained is the one that can be seen in Figure 5. Then, applying Equation (A2) for three components and second grade interactions, the result obtained was:

(1) $$\frac{(q+m-1)!}{m!\cdot (q-1)!}=\frac{(3+2-1)!}{2!\cdot (3-2)!}=6$$

The result pinpoints the number of mixtures that had to be explored. Each sample of the simplex-lattice design was simulated with Geant4, but only the three black-dotted samples (a–c, in Table 2) had been prepared in laboratory. The three remaining samples: (d–f) provide low shielding from hard X-rays, but are still interesting for applications involving low energies (0–60 keV) and requiring good mechanical resistance to strain.

### 2.4. Direct Radiography

**Figure 6:**
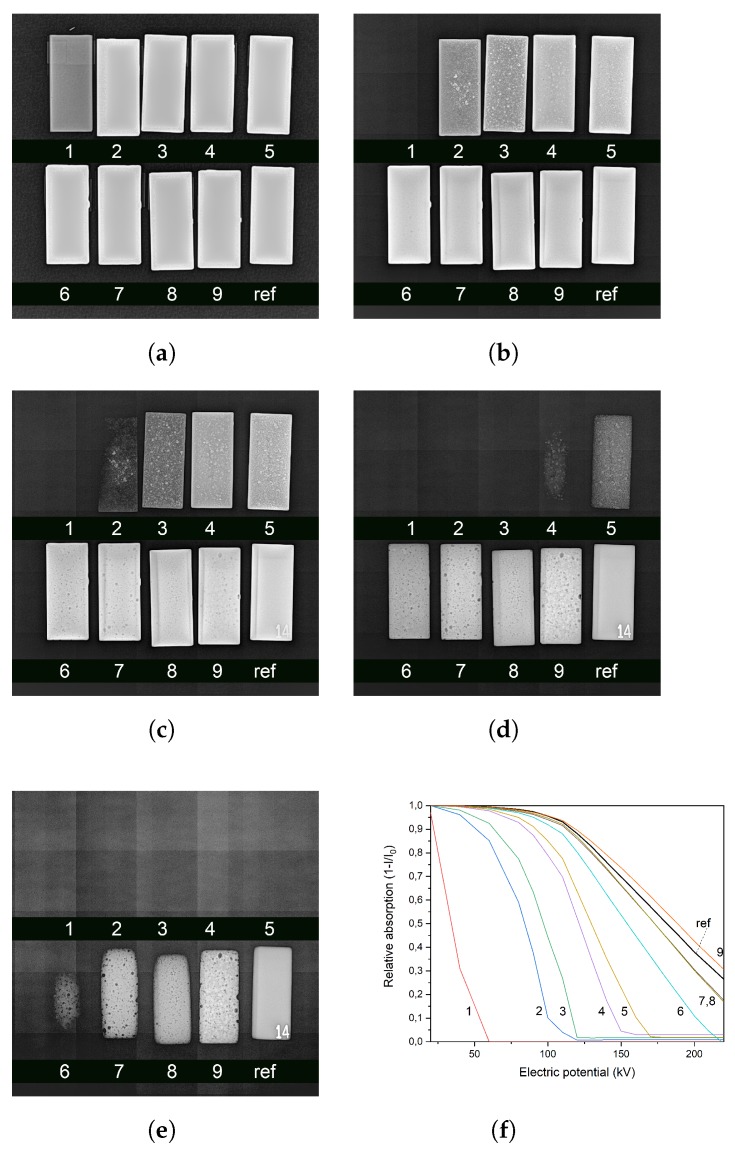
Radiographies of epoxy-barite samples at increasing percentage *w*/*w* in BaSO_4_. Samples are labeled as in Table 2. Ref sample is the reference of steel. In (**f**), increasing X-ray absorbance profiles can be osberved, from left (0% *w*/*w* barite) to right (85% *w*/*w* barite). Greyscale data associated to (**f**) and relative statistics are available as supplementary material. (**a**) 20 kV; (**b**) 60 kV; (**c**) 100 kV; (**d**) 150 kV; (**e**) 220 kV; (**f**) Profile comparison.

**Figure 7:**
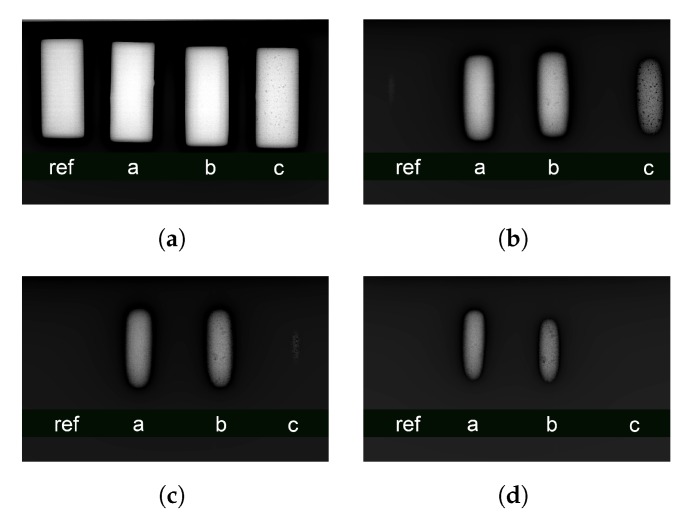
Radiographies on the three ternary mixtures (samples a, b, c in Table 2) developed in laboratory compared with the same steel sample for reference. In this case the photon flux was raised by increasing the current intensity to explore the behavior of the screens in a more severe experimental condition. (**a**) 120 kV; (**b**) 180 kV; (**c**) 200 kV; (**d**) 220 kV.

Figure 6 shows the radiographies collected over the nine first-generation samples, plus a reference made of steel, positioned on the bottom-right corner of the images. A graph of the performances of each sample compared to steel can be observed in Figure 6f. The curves were obtained by considering the gray scale values of the samples in the picture. The data were obtained by calculating the average gray scale value (from 0 to 255) over the whole sample area and then calculating the relative absorption. Curve 9, referred to the sample 85% in weight of barite, overperforms steel in terms of radiation absorption, a property that can be appreciated also by eye in Figure 6e. This result apparently clashes with the results in Figure 1, which instead shows that at 220 keV steel should be a better screen than this mixture. The reason of these discrepancies resides once again in the structure of the first simulation, which involved a monochromatic source of X-rays, which is not the real-world case, in the radiographic system. Also, a generator on which is applied an electric potential of 220 kV, provides just few photons with energy of 220 keV; it has instead a maximum of the emission band at one third of the supplied tension (which gives about 70–75 keV, a range where the Epoxy-BaSO_4_ composite effectively performs better than steel). Another information that comes from these images resides in the high defectivity ratio that characterize the most additivated samples. These defects are mainly air bubbles that remain trapped in the highly viscous matrix. Voids represents points in the samples in which the optical path for the photons is shorter compared to the rest of the bulk. Also, this kind of defect lowers the resistance of the samples in terms of tensile strength. The second set of simulations with Geant4 suggested that the second generation of samples would be more radiopaque than the previous ones due to the presence of bismuth oxide, therefore the setup and the experimental conditions were changed increasing the flux and detector sensitivity. Therefore, a direct comparison between the two results could not be made. In Figure 2 it can be observed that all samples strongly overperform steel, which is fully transparent at 180 kV. In the same image, sample *c* shows a large amount of bubbles in the bulk. As foretold by the hard simulations, an increase of bismuth oxide corresponds to a higher screening performance of the shields (Figure 7).

### 2.5. Mechanical Characterization

**Figure 8:**
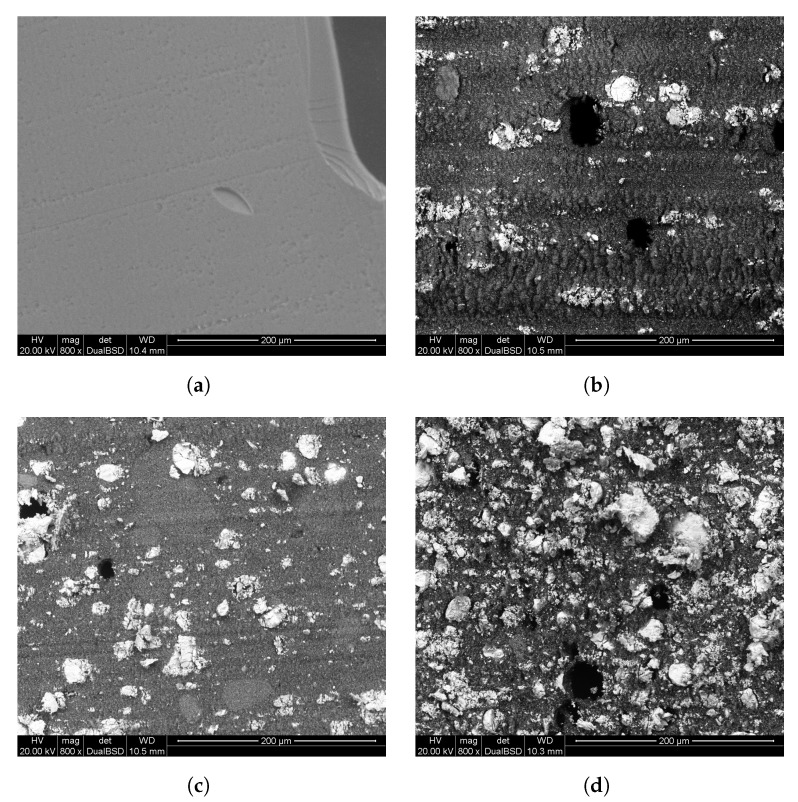
The four pictures show the sample section surface after the tensile test. The large Bi_2_O_3_ domains, visible in Figure 8d, are the main cause of fracture of the samples, while in Figure 8b, it can be seen that barium sulfate has a finer grain size. Figure 8c shows sample b, which has few large inorganic domains and a higher volumetric ratio of epoxy resin. (**a**) Epoxy resin; (**b**) Sample a; (**c**) Sample b; (**d**) Sample c.

To evaluate the mechanical performances of the composite samples, a study of stress/strain properties was done. Five dog bone shaped samples suitable for the tensile tests were obtained for each mixture of the second generation series of samples. Another five samples of pure epoxy resin were prepared and used as a reference. These samples were subject to traction until break point. Data reported in Table 3 refer to each mixture performances under stress. The pure epoxy resin, showed a plastic behavior, with plastic deformation before the break point. This was not observed for mixture samples, which showed fragile behavior and lower resistance if compared to the pure epoxy resin. The sections of the fracture point were observed with SEM (Figure 8), which showed, in additivated samples, micrometric domains of inorganic powder which are responsible for the fracture. The SEM images evidenced an increase of the size of bismuth oxide domains in the matrix proportional to the increase of its amount in the sample. This agglomeration (particle segregation) phenomenon is related to the large uncertainty on the strain resistance of the sample *a*. On the contrary, barium sulfate is finely dispersed in the sample, but having a lower density than bismuth oxide, the overall inorganic percentage by volume in sample *c* is higher than the percentage in the *a* sample. This explains well also the high uncertainty and the low strain resistance of this sample. Sample *b* has the best mechanical performances because of the low amount of bismuth oxide, whose big grains are the main initiators of the fractures, and an acceptable volume ratio of powders over the resin. In the same images bubbles, which developed during the polymerization and were not expelled from the matrix due to the high viscosity of the samples, can be observed.

### 2.6. LCA Study

**Figure 9:**
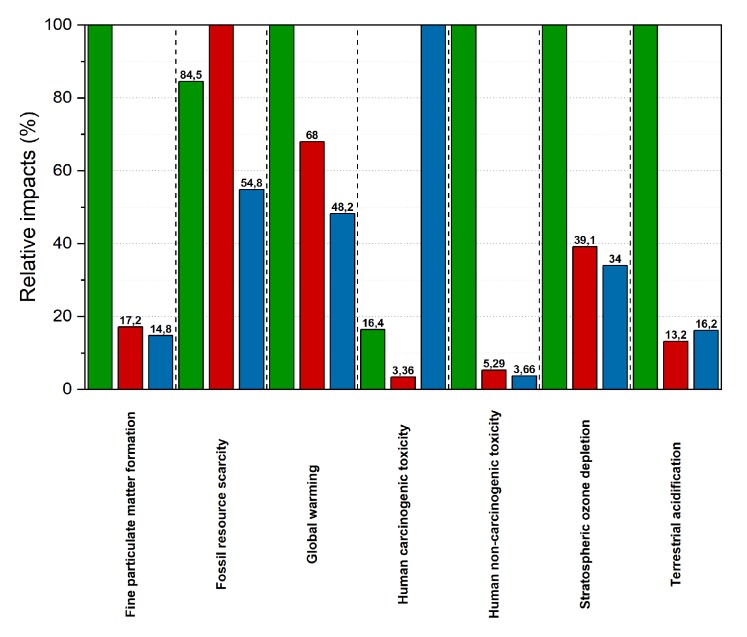
The histogram is an extract from the previous table which shows the relative impacts for the main and most reliable factors showed in Table 4. Data were normalized to the maximum value for a rapid evaluation of the advantages and disadvantages of each material. Green bars refer to the impacts calculated for lead, red bars for the composite samples and blue bars are referred to steel impacts.

A “from cradle to gate” LCA calculation was performed to assess the environmental performances of the produced epoxy-additive composites. Then, the results were compared with traditional screens made of lead and stainless steel. The LCA model was built using the openLCA software tool as described in Appendix D. The release 3.5 of the ecoinvent database was used as a reference for all the background data included in the LCA model. Foreground inventory data about raw materials, energy and processes, used for the making of the epoxy-additive samples, were taken directly from formulations while inventory data about the “from cradle to gate” life cycle of both screens of lead and stainless steel were taken from the literature. In this calculations a wide set of factors were considered and Life Cycle Impact Assessment results can be seen in Table 4. For reference, just sample *b* was compared to the traditional screens. This sample was selected among the three mixtures developed because of its intermediate screening properties, costs, environmental and human impact. Choosing just one of the three simplex samples does not heavily affects the following calculations, as the three epoxy samples were previously compared between them, and they showed little differences in the impacts. The variation of 20% in weight of Bi_2_O_3_ between the simplex samples does not affect the results heavily since the main reason due to the change in the impact relies in the relative scarcity of bismuth minerals and the consequent ore extraction. This process, compared to barium minerals, which instead are very common, is the main reason for the higher impact of bismuth on the calculation. Except for fossil resource scarcity, composite shielding materials are more sustainable than lead, and in terms of carcinogenicity they take a huge advantage over steel, as shown in Figure 9. In Table 4, impacts referring to the composite samples are generally lower than the ones referred to lead, except the parameters that depend on the quantity of the source ores (fossil resource scarcity, mineral resource scarcity...). Stainless steel impacts, compared to the composite ones are generally lower. However, human carcinogenic toxicity of steel is strongly higher (two orders of magnitude) than that of the samples described in this work.

## 3. Discussion

This study identified mixtures of composite materials for X-ray shielding that have low toxicity for human health, low environmental impact as assessed by LCA analysis, low costs of production and processing and good mechanic resistance. Simulations through Geant4 of pure materials from scientific literature, showed in Figure 1b, allowed to identify the best performing materials in X-ray shielding. The chosen additives were bismuth oxide and barium sulfate. BaSO_4_ was chosen because it has similar performances in shielding to zinc oxide, but its cost is about 100 times lower. Nine BaSO_4_ - Epoxy composites in increasing weight percentage of additive were prepared and the simulations made with Geant4 were confirmed. Moreover, the upper limit of mixability between the resin and the inorganic additive was tested resulting in 85%w/w of additives. Bismuth, which showed excellent shielding properties in simulations, was selected to be mixed with barium sulphate to obtain high performances mixtures. The simplex-lattice experimental design for mixtures of Figure 5 was then developed, identifying each of the three components with a predominant factor (high screening properties, high stress resistance and low costs). Of the six resulting mixtures, only three were produced in laboratory, as the main requested property was the high x-ray absorption. The three others mixtures were not prepared because the observed transmission factors resulting from the simulations were too high for being integrated as industrial shielding materials. These samples can still be applied where the energies of produced X-rays are lower than 50 keV (such as the medical applications) and the strain resistance required has to be upper than 13–15 MPa.The three simplex mixtures were then analyzed through direct radiography and showed a screening effect trend in agreement with Geant4 prediction. Produced samples overperformed steel in terms of radiopacity with a much easier formability and workability and weight reduced up to the one third of a steel screen. It was thus possible to develop composite formulations depending in shielding performance, weight and costs requirements: mixture *c* is suitable as steel replacement. For systems that involve higher energies, mixtures *a* and *b* are the best choice. For other applications, like the medical ones, where energies involved are lower than 50 keV, the three remaining mixtures (d,e,f) are well suited. Looking to the molecular level, it was not possible obtaining direct indications about the interaction and the adhesion between the filler and the matrix. On one hand, the composites showed sufficient mechanical properties also at high loadings and this suggests that some polymer/additive interaction occurred. On the other hand, the aggregation of the additive suggest that it should be modified to improve its interaction with the epoxy matrix to improve the adhesion and thus optimizing both dispersion and mechanical properties. This can be done by improving the hydrophobicity of the additive surface by binding an organic molecule such as stearic acid or sodium dodecylsulfate. This approach will be studied and developed in a future work.

## 4. Conclusions

In this study, different formulation for easily formable composites lightweight shielding materials were proposed. The work explored the best performing mixtures between bismuth oxide, barium sulfate and epoxy resin, using a simplex-lattice experimental design for mixtures. The resulting mixtures (see Section 2.3 for details) can be used in different fields based on the requirements of shielding ratio, mechanical properties and lightness. For low photon-energy applications, such as the medical field, 20:20:60, 20:40:40 and 40:20:40 BaSO_4_/Bi_3_O_3_/epoxy resin w/w mixtures are the best ones, due to their lightweight and good performances in shielding and mechanical resistance. For hard X-ray industrial applications, such as radiography and computed tomography with energies in the 20-220keV range, the best performing mixtures are: 60:20:20, 40:40:20 and 20:60:20 BaSO_4_/Bi_3_O_3_/epoxy resin w/w. In details, three mixtures (labeled *a*–*c*) have performances higher than steel (mixture *a* has 4.5% more shielding capability, mixture *b* has 23.4% and mixture *c* has 33.1% with a weight reduction ranging from 55% to 62% depending on which mixture is chosen), becoming good candidates for traditional screen replacement. Besides physical, chemical, morphological and mechanical characterization to demonstrate their suitability for real world usage, an LCA study was conducted to compare screens made of lead and steel with the produced mixtures. Results show that the produced samples have an overall environmental impact lower than those based on lead (Global warming 32% less, fine particular matter formation 82.8% less, human non-carcirogenic toxicity 94.71% less,…) and a lower human carcinogenic impact (96.64% less) than those based on steel.

## 5. Materials and Methods

### 5.1. Materials and Preparation

All samples were prepared starting from technical grade reagents. A two-components epoxy resin was purchased by S.E. Special Engine S.r.l. (Torino, Italy). Component A (Sepox 225) of the epoxy was made up by 80%w/w of bisphenol-A-(epichlorhydrin) and epoxy resins with average molecular mass lower than 700 Dalton. Component B (DK 505) was 35%w/w of 3-aminomethyl-3 5 5-trimethylcyclohexylamine and between 18%w/w of polyoxypropylenediamine.

Barite ($85\%\;w/w\;{\mathrm{BaSO}}_{4},0.8\%\;w/w$ calcite, 13.6%w/w dolomite) was given by Itaprochim (Garlasco, Italy) and other two samples of Barite (Blanc Fixe JM3B and Blanc Fixe G) were given by Universal Services S.r.l. (Milano, Italy) and produced by Solvay (Massa, Italy), Bi_2_O_3_ (>99% w/w) and SnO_2_ (>99% w/w) was given by Todini (Monza, Italy) and Thermo Fischer (Kandel, Germany). Samples were prepared as described in Appendix C.

### 5.2. Analysis Methods

Powder materials were analyzed through XRPD and the diffraction patterns were compared with the ones simulated with the corresponding .cif files downloaded from the Crystallographic Open Database (COD) [15]. PXRD pattern were collected with a Thermo ARL XTRA48 X-ray diffractometer. Patterns were collected in the 2θ angle range from 15 to 65 degrees. X-ray tube’s electric potential was set to 45 kV and current intensity to 40 mA. Slits were set as a standard measurement 2 mm, 4 mm, 0.5 mm, 0.2 mm. Resolution was set to 0.02° and scan speed to 2.00°/min. Radiographic tests for sample of binary mixture BaSO_4_/Epoxy were performed by the direct digital radiography technique; an Yxlon Y.XMB X-ray module was used as X-ray source and a Carestream DRX-1 as detector (filament current: 10 mA; voltage: 20 kV–220 kV). In the case of simplex samples, the computed digital radiography technique was used with the same previous X-ray source and a GE CRxFlex IPS imaging plate as detector (current: 5 mA; voltage 20 mA–220 kV). Radiographies collected were processed with ImageJ to extract grey-scale values for confrontations. Sections 10 μm in thickness were cut on a Leica RM 2125 RT rotary microtome; SEM images at different magnification were recorded on a Quanta 200 FEI Scanning Electron Microscope equipped with EDAX EDS attachment, using a tungsten filament as the electron source at 20 kV. All samples were coated with an Au layer of 20 nm to prevent surface charging.

### 5.3. Modeling

#### 5.3.1. Geant4 Simulations

Simulations of radiopacity were performed (on a Sony Vaio laptop 4 GB RAM, 1.4 GHz i7 processor with Ubuntu 18.10) by Geant4. The simulations were performed in two steps: a first approach with monochromatic X radiation and a second one considering the generation of a Bremsstrahlung spectrum of tungsten as source for the sample irradiation, as described in Appendix B. (By using Geant4, a first screening of materials was conducted over the different solution found in the scientific literature. The simulation setup was designed by creating a 1 cm sided cube of material irradiated along the *X*-axis with a monochromatic beam of X-rays counting $10^{4}$ photons. The experiment was repeated at steps of 25 keV in the range of 0–350 keV so that the best performing materials could be selected).

#### 5.3.2. openLCA Simulations

For this project the open source professional solution named openLCA 1.9 and the ecoinvent 3.5 database were used. openLCA by implementing powerful and flexible ways to model life cycle systems, with the ability to calculate environmental, social and economic indicators, with plugins which are providing different more specific elements, and with its open architecture which eases import and export of data and integration in other IT landscapes, is the free and open source software for modeling the life cycle of things [16]. The ecoinvent database is the world’s leading LCI database which delivers both in terms of transparency and consistency [17]. 

## Figures and Tables

**Table 1:** Samples of BaSO_4_—Epoxy composites to assess additive amount effect.

BaSO_4_ Samples		
Sample Name	BaSO_4_ Weight %	Epoxy Weight %
1	0	100
2	30	70
3	40	60
4	50	50
5	60	40
6	70	30
7	80	20
8	80	20
9	85	15

**Table 2:** Mixtures provided by Simplex-lattice design.

BaSO_4_–Bi_2_O_3_ Samples			
Sample Name	BaSO_4_ Weight %	Bi_2_O_3_ Weight %	Epoxy Weight %
a	20	60	20
b	40	40	20
c	60	20	20
d	40	20	40
e	20	20	60
f	20	40	40

**Table 3:** Each mixture was subject to tensile testing with five replicas for each sample. Pure epoxy resin samples were tested as a reference. The data show that epoxy resin would break in the range 26–305 MPa after plastic deformation. Samples *a* and *c* show a low tensile resistance, accompanied with a huge variation coefficient. Sample *b* has instead a good resistance and a low variation coefficient, suggesting a very good reproducibility.

Sample	Average Stress at Break Point	Standard Deviation	Variation Coefficient %
Epoxy resin	28.68 MPa	±1.98 MPa	6.91%
*a*	9.654 MPa	±5.34 MPa	55.30%
*b*	14.12 MPa	±1.19 MPa	8.46%
*c*	5.06 MPa	±1.62 MPa	31.99%

**Table 4:** LCA study results using the ReCipe 2016 method. The comparison was made among lead, steel and the average composite sample (*b*), since a comparison between the three developed mixtures would show very similar impacts.

Impact Category	Composite	Lead	Steel	Unit
Fine particulate matter formation	$3.0083\times 10^{-3}$	$1.7537\times 10^{-2}$	$2.5890\times 10^{-3}$	kg PM2.5 eq
Fossil resource scarcity	$7.6487\times 10^{-1}$	$6.4664\times 10^{-1}$	$4.1914\times 10^{-1}$	kg oil eq
Freshwater ecotoxicity	$8.4606\times 10^{-2}$	$9.1613\times 10^{-1}$	$9.3025\times 10^{-2}$	kg 1,4-DCB
Freshwater eutrophication	$6.1143\times 10^{-4}$	$5.4034\times 10^{-3}$	$6.4810\times 10^{-4}$	kg P eq
Global warming	2.0190	2.9701	1.4331	kg CO_2_ eq
Human carcinogenic toxicity	$7.7873\times 10^{-2}$	$3.8059\times 10^{-1}$	2.3177	kg 1,4-DCB
Human non-carcinogenic toxicity	2.3725	$4.4880\times 10^{1}$	1.6433	kg 1,4-DCB
Ionizing radiation	$1.5145\times 10^{-1}$	$1.0113\times 10^{-1}$	$2.8174\times 10^{-1}$	kBq ^60^Co eq
Land use	$1.9765\times 10^{-2}$	$2.8625\times 10^{-2}$	$7.5114\times 10^{-3}$	m${}^{2}$a crop eq
Marine ecotoxicity	$1.0682\times 10^{-1}$	1.2924	$1.3559\times 10^{-1}$	kg 1,4-DCB
Marine eutrophication	$2.8346\times 10^{-4}$	$1.2057\times 10^{-4}$	$4.5165\times 10^{-5}$	kg N eq
Mineral resource scarcity	1.7336	$5.0973\times 10^{-1}$	$2.1350\times 10^{-3}$	kg Cu eq
Ozone formation, Human health	$6.0554\times 10^{-3}$	$1.6108\times 10^{-2}$	$3.4478\times 10^{-3}$	kg NOx eq
Ozone formation, Terrestrial ecosystems	$6.3136\times 10^{-3}$	$1.6403\times 10^{-2}$	$3.5129\times 10^{-3}$	kg NOx eq
Stratospheric ozone depletion	$1.1278\times 10^{-6}$	$2.8819\times 10^{-6}$	$9.7955\times 10^{-7}$	kg CFC11 eq
Terrestrial acidification	$7.1376\times 10^{-3}$	$5.4225\times 10^{-2}$	$8.7846\times 10^{-3}$	kg SO_2_ eq
Terrestrial ecotoxicity	7.2130	$4.9729\times 10^{1}$	$1.0801\times 10^{1}$	kg 1,4-DCB
Water consumption	$3.5767\times 10^{-2}$	$2.7976\times 10^{-2}$	$2.6284\times 10^{-2}$	m${}^{3}$

**Figure A2:**
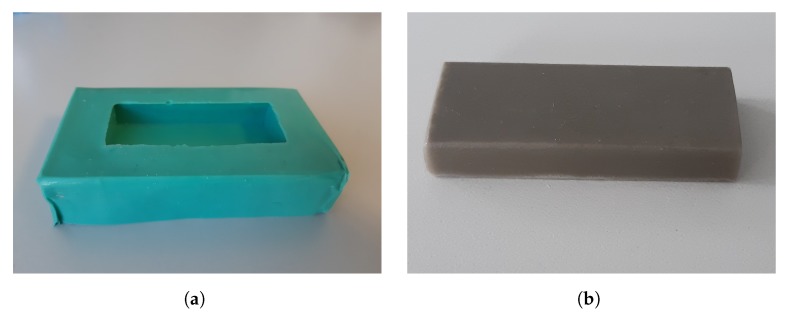
In the image on the left, a photo of a silicon rubber mold that was used for the production of the radiopaque samples. In the image on the right, a photo of one of the produced samples. (**a**) Silicon rubber mold.; (**b**) 30% *w*/*w* BaSO_4_ sample.

**Figure A3:**
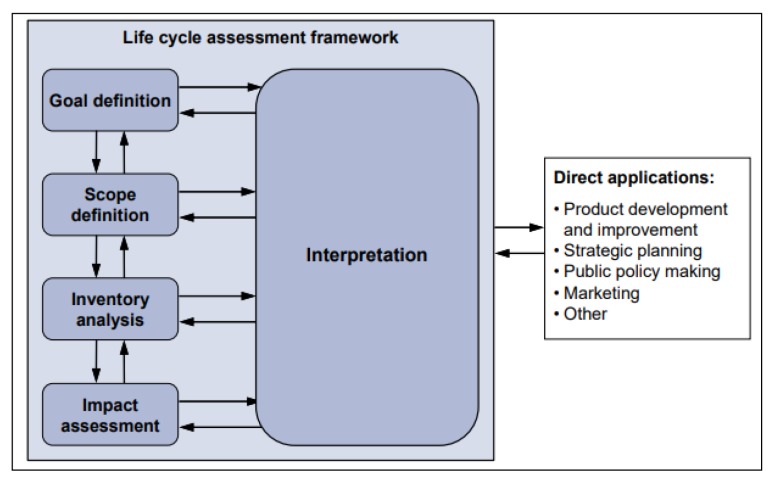
Framework for life cycle assessment (from ISO 14040:2006; modified) [22].

**Figure A4:**
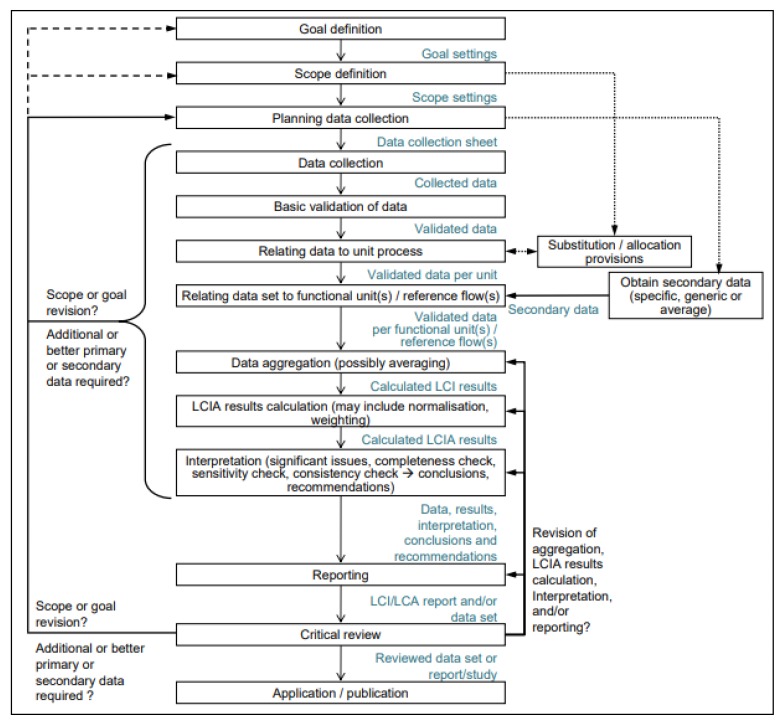
Details of the iterative approach to LCA, with focus on inventory data collection and modelling (from ISO 14044:2006, modified) [22].

**Figure A5:**
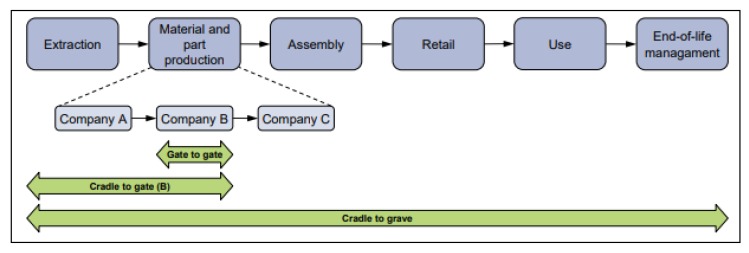
Types of LCA studies classified by system boundaries taken into account [22].

## Supplementary Materials

Supplementary Materials are available online at https://www.mdpi.com/1422-0067/21/3/833/s1.

## Appendix A. Design of Experiments (DOE)

Experimental design consists in planning experiments in order to obtain the maximum information contained in a system with the least experiments possible. Even if the construction of a regression model is not the aim of a series of experiments, using experimental designs is common practice to explore an experimental domain in the most complete way. Mixtures, however, require more attention than other studied systems, mainly because the properties of the product depends on the relative quantity of the components introduced in the mixture, not on their absolute quantity. This leads to a consequence: none of the components can vary independently, because the sum of the relative quantities of each mixture has to be equal to 1.

(A1) $$\sum_{i=1}^{q}x_{i}=1$$

Equation (A1) represents the said constraints to the mixture, that prevents the use of common experimental designs such as Factor Designs. The three components of the studied mixture: epoxy resin, BaSO_4_ and Bi_2_O_3_ can be associated with properties of the final samples, respectively: mechanical resistance, low costs and best radiopacity. Mixing these three components would lead to a compromise between these three properties, directly connected to the chemicals. To explore this experimental domain, a *Simplex-lattice design* for three components and two as maximal degree of the theoretical model was studied. These kinds of DOE are component-independent and are generated only from the algorithm:

(A2) q+m−1m=(q+m−1)!m!·(q−1)!

which leads to six experiments. Mixtures are then defined, for each component, by the series:

(A3) $$x_{i}=0,\frac{1}{m},\frac{2}{m},\frac{3}{m},\ldots 1$$

which respectively represent the vertexes of an equilateral triangle and the midpoint of each side, as represented in Figure 5. These experiments make up a *simplex* which will be the explored part of the domain.

## Appendix B. Geant4 Simulations

To avoid wasting materials and optimize the experiments, different sets of simulations were performed by using Geant4: a toolkit for the simulation of the passage of particles through matter with Monte Carlo methods [18,19].

Once defined the experimental domain by consisting in BaSO_4_, Bi_2_O_3_ and epoxy resin, a simplex mixture design was chosen to explore it and the six samples of the experimental design (see Figure 5) had to be more accurately simulated by reproducing the phenomenon that occurs in the radiographic cabin. For these samples, the geometry of the simulation changed. A target of tungsten of 300μm of thickness was positioned between the radiopaque screen and the source of the beam, which also had been changed into an electron beam of $10^{6}$ units. Electrons travel for 10 cm in vacuum until they hit the target. The impact generates an emission of the characteristic X-ray spectrum of W (Bremmstrahlung spectrum and characteristic X-rays emissions), which is then focused on the shield sample. Steel was also simulated for reference. Collected data were then plotted and only best-performing samples in radiopacity were then produced.

## Appendix C. Sample Preparation

To have reproducible samples in shape and sizes, silicon rubber mold was prepared. The mold was made of a Room-Temperature-Cured (RTC) two component silicon rubber, which was supplied by S.E. Special Engine S.r.l. The components, DD Rubber A and DD Rubber B were mixed together in 1:1 ratio and outgassed for 10 min to eliminate bubbles from the rubber bulk. Steel bars of the same size of the desired samples were glued to a baking tray in order to obtain the negative of the mold. Silicon rubber mixture was poured into the tray and was outgassed for another 10 min. And after three hours of curing at room temperature (RT), the mold was ready to be used Figure A2a. Since the epoxy resin does not adhere to silicon the mold could be used many times.

Epoxy/inorganic additive composites were prepared dispersing the inorganic additive in Sepox 225 and then adding DK_505_. The ratio between Sepox 225 and DK_505_ has to be 2:1. The mixture with the two components and the additive was then stirred for 10 min and then outgassed. Then it was cured at RT for 24 h, plus two hours at 60 °C. In highly loaded samples, the stirring could be not performed automatically and they were stirred by hand. As an example, the sample 30%w/w in BaSO_4_ is shown in Figure A2b.

## Appendix D. Life Cycle Assessment

Life Cycle Thinking (LCT) and Life Cycle Assessment (LCA) are the scientific approaches behind modern environmental policies and business decision support related to Sustainable Consumption and Production (SCP). Life Cycle Assessment (LCA) is a structured, comprehensive and internationally standardised method. It quantifies all relevant emissions and resources consumed and the related environmental and health impacts and resource depletion issues that are associated with any goods or services (“products”). Usually, Life Cycle Assessment takes into account a product’s full life cycle: from the extraction of resources, through production, use, and recycling, up to the disposal of remaining waste. Critically, LCA studies thereby help to avoid resolving one environmental problem while creating others: This unwanted “shifting of burdens” is where you reduce the environmental impact at one point in the life cycle, only to increase it at another point. Therefore, LCA helps to avoid, for example, causing waste-related issues while improving production technologies, increasing land use or acid rain while reducing greenhouse gases, or increasing emissions in one country while reducing them in another. Life Cycle Assessment is therefore a vital and powerful decision support tool, complementing other methods, which are equally necessary to help effectively and efficiently make consumption and production more sustainable.The ISO 14040 [20] and 14044 [21] standards provide the indispensable framework for Life Cycle Assessment (LCA) (see Figure A3).

To carry out an LCI or LCA study is almost always an iterative process: once the goal of the work is defined, the initial scope settings are derived that define the requirements on the subsequent work. However, as during the life cycle inventory phase of data collection and during the subsequent impact assessment and interpretation more information becomes available, the initial scope settings will typically need to be refined and sometimes also revised. Figure A4 gives a more detailed overview of the iterations [22].

Depending on the stages of the life cycle that the study analyzes, three different types of LCA studies can be identified: cradle to grave, cradle to gate, gate to gate (see Figure A5).

Since the building of an LCA model is a quite often complex operation which requires to define and connect the existing network of relationships between processes that belongs to different stages of their life cycle, a software tool and a reference database to which refer environmental data that are not directly collectable, are commonly used. A software tool helps and allow the LCA modeler to speed up the calculation and to have some features available to better interpret the results of the assessment while a comprehensive database provides well documented process data for different families of products and helps the LCA modeler to make truly informed choices about their environmental impact. References
